# Preventive Evolutionary Medicine of Cancers

**DOI:** 10.1111/eva.12033

**Published:** 2012-12-05

**Authors:** Michael E Hochberg, Frédéric Thomas, Eric Assenat, Urszula Hibner

**Affiliations:** 1 ISEM, UMR5554 CNRS/UM2/IRD, Université Montpellier 2Montpellier, France; 2 The Santa Fe InstituteSanta Fe, NM, USA; 3 CREEC, Université Montpellier 2Montpellier, France; 4 MIVEGEC, UMR 5290 CNRS/IRD/UM1Montpellier, France; 5 IGMM, UMR5535 CNRS/UM2Montpellier, France

**Keywords:** cancer, chemotherapy, evolution, evolutionary cell biology, lifestyle, microenvironment, preventative evolutionary medicine, prevention

## Abstract

Evolutionary theory predicts that once an individual reaches an age of sufficiently low Darwinian fitness, (s)he will have reduced chances of keeping cancerous lesions in check. While we clearly need to better understand the emergence of precursor states and early malignancies as well as their mitigation by the microenvironment and tissue architecture, we argue that lifestyle changes and preventive therapies based in an evolutionary framework, applied to identified high-risk populations before incipient neoplasms become clinically detectable and chemoresistant lineages emerge, are currently the most reliable way to control or eliminate early tumours. Specifically, the relatively low levels of (epi)genetic heterogeneity characteristic of many if not most incipient lesions will mean a relatively limited set of possible adaptive traits and associated costs compared to more advanced cancers, and thus a more complete and predictable understanding of treatment options and outcomes. We propose a conceptual model for preventive treatments and discuss the many associated challenges.

## Introduction

Despite increasing cancer incidence due largely to ageing populations and improved diagnosis, the cancer-related mortality in industrialized countries is decreasing (Howlader et al. 2012). This undeniable victory in the ongoing war against cancer, declared in 1971 through the National Cancer Act (http://legislative.cancer.gov/history/phsa/1971), is mainly due to improved prevention and better detection of early neoplasms. The preventive measures include changes in lifestyle, pressure to abandon dangerous habits like cigarette smoking or excessive alcohol consumption and efficient campaigns aimed at eradication of infections strongly associated with many common cancers (e.g. de Martel et al. 2012).

Early detection of primary tumours or precancerous lesions followed by aggressive therapy has led to considerable improvement of prognosis in some types of cancer, such as colorectal carcinoma (http://globocan.iarc.fr/fact sheets/cancers/colorectal.asp). The benefits of widespread screening for breast and prostate tumours are more controversial. Indeed, it is not clear how much of the observed decrease in mortality caused by these common cancers is due to early detection as opposed to improved treatment options (Bulliard and Levi 2012). Moreover, massive screening of populations at risk has given rise to overtreatment of patients carrying lesions that would most probably never develop into aggressive carcinomas (e.g. Welch and Black 2010; Klotz 2012).

There are several explanations for these mixed results, which are costly in terms of both health care and human suffering. First, we do not have a sufficient understanding of tumour biology or interactions with the host to reliably predict which lesions will progress to malignancy and which will be prevented from expanding or even be eliminated by the host (Folkman and Kalluri 2004; Bissell and Hines 2011). Second, although most tumours detected by screening programmes are small compared with late-stage tumours, they are already populous enough to contain numerous subclones, of which some are likely to be resistant to any treatment other than physical removal. Indeed, even tumours that measure as little as 1 mm^3^, which would escape detection by all but the most efficient ultra-high-resolution analysis (e.g. Wallace and Kiesslich 2010), contain about 10^6^ transformed cells and are the product of at least 20 cycles of cell division (assuming a binary tree and ignoring the common events of apoptosis and senescence in the growing tumour). Given the high rate at which mutant cells are produced in at least some tumours (Loeb 2011) and associated genetic instability (considered an enabling characteristic for several of the cancer hallmarks, Hanahan and Weinberg 2011), this allows ample opportunity for the emergence of heterogeneous cell populations containing chemoresistant clones in neoplasms prior to their detection.

Here, we propose that ecological and evolutionary insights can be used to design more efficient interventions for cancer prevention and management. The central tenet of this framework is that protecting and bolstering natural defences through lifestyle changes and/or active medical treatments implemented *before* any clinical signs (either tumours or disease symptoms) are manifested has a firm ecological and evolutionary basis as an alternative to classical therapies, at least for certain risk groups and for certain cancer types. We present a conceptual model for such evolutionary prevention of cancer, and argue why this should constitute a major effort in health programmes and medicine.

## What is the added value of evolutionary thinking for understanding cancer?

Evolution provides a framework for understanding the interrelations among the environment, (epi)genotypes and phenotypes and how they influence tumour phenology. The evolutionary process in cancer occurs at two characteristic scales: emergence at the population level and progression within individual hosts.

### Cancer emergence

**Figure 1:**
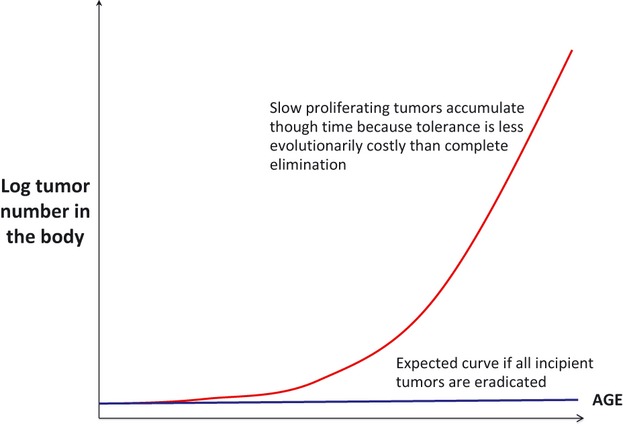
Hypothesis that natural selection promotes mechanisms that hold tumours in check. Due to energetic costs and constraints involved in completely eradicating emergent neoplasms, natural selection will control tumours until ages at which effects on Darwinian fitness are insufficient. We predict that innocuous lesions should therefore accumulate through time, with some of them becoming full-blown cancers at older ages shielded from natural selection.

Evolutionary theory predicts the clinically observed increase of cancer incidence with age (e.g. Balducci and Ershler 2005). However, many if not most people at or beyond reproductive age carry *in situ* tumours, but do not develop cancer (Folkman and Kalluri 2004; Bissell and Hines 2011). This observation illustrates the notion that multicellular organism defences curb or eliminate most cancerous lesions, or at least limit their proliferative tendency. In other words, natural selection has favoured the evolution of protective mechanisms against cancer before or during reproductive ages (Crespi and Summers 2005; DeGregori 2011). More speculatively, these mechanisms may not involve complete lesion or tumour elimination, because the costs of such adaptations might exceed the benefits at ages when natural selection is most intense. Interesting parallels exist with infectious diseases: avoidance and tolerance of damage caused by pathogens and parasites are frequent in nature (Medzhitov et al. 2012), and it has been suggested that the majority of host defences that have arisen during evolution are tolerance mechanisms that control damage rather than eliminate an enemy (Read et al. 2008). For cancers, these considerations mean that organisms should hold tumours in check, but not necessarily eliminate them when too costly to reproductive fitness (Fig. 1). If correct, then we would predict that innocuous lesions should accumulate through time, with some becoming cancers once an individual reaches an age (i.e. reproductive or postreproductive) where natural selection was less efficient at retaining mechanisms for tumour suppression. It is currently unclear to what extent this explains the incidence of clinical cancers, but consistent with our hypothesis, there is increasing evidence that some solid tumours may take decades to emerge (e.g. Jones et al. 2008; Yachida et al. 2010).

### Within-host progression and chemoresistance management

The evolutionary perspective encompasses two pervasive population phenomena (i) (epi)genetic changes are very likely to be composed of both advantages (higher population growth) and disadvantages (costs), and (ii) the net fitness effect and thus potential to survive and spread will depend on (changing) environments. Because cancer growth is usually associated with increased genotypic and phenotypic heterogeneities (e.g. Clappier et al. 2011; Gerlinger et al. 2012), we would expect that assessing balances between costs and benefits of different acquired cancer hallmarks be more straightforward in nascent tumours compared with those that are more advanced. Together with the expectation that chemoresistant cells have higher probabilities of occurrence with time postemergence, this has important therapeutic implications: the evolutionary framework provides the reasons for why early treatment can be directed at tipping the balance from net benefits to net costs in early lesions, with the dual objectives of slowing or curbing cancer progression and preventing the emergence of chemoresistance due to cellular proliferation (see also Maley et al. 2011). An important insight from evolution is that resistance to chemotherapies needs to be managed in the same way that, for example, invasive species, infectious diseases or agricultural pests are controlled, so as to slow or prevent the emergence of resistant strains and associated population resurgence (e.g., Vacher et al. 2003; Gatenby et al. 2009; Read et al. 2011).

## Preventive evolutionary medicine of cancers

Given the emergence of novel mutants in a growing cell population, the genetic instability and the numerous epigenetic alterations associated with many detectable and (arguably) most advanced cancers, medicine will rarely, if ever, predictably manage late-stage disease. We suggest that knowledge about these same evolutionary factors promoting the emergence of chemoresistant lineages – i.e. cell population turnover and tumour heterogeneity – can be used to develop approaches to *prevent* cancer in the first place.

Two characteristics of cancer give encouragement for investigating preventive strategies. First, cancers are thought to proceed through a number of premalignant states before they are diagnosed (Vogelstein and Kinzler 1993; Fang et al. 2008; Gatenby and Gillies 2008). While there is some predictability in this progression at least for certain cancer types, considerable research will still be necessary to delineate combinations of genetic and epigenetic alterations and phenotypic changes associated with malignancy and metastasis. Second, what often prevents chemotherapies from eradicating cancers is a patient's limited tolerance to the most appropriate drugs, and this is particularly a problem in older patients and those weakened by late stage cancers.

We propose that the logical consequence of ecological and evolutionary thinking is that undiagnosed, incipient cancers in individuals at risk should be the primary targets of a global health policy, as this is the most likely means for either eradicating malignancies before chemoresistance appears or managing cancer growth when eradication is not possible. The idea of early detection as a means of prevention is of course not new and is already put into practice. However, as already discussed, even early detection typically uncovers tumours that are already phenotypically heterogeneous. Furthermore, while screening programmes efficiently detect many early cancerous and precancerous lesions, they are often rather poor predictors of which of those will subsequently develop into a malignant tumour, thus considerably lowering the usefulness of many of the expensive, time-consuming and stress-generating screening programmes.

Understanding the limits of natural selection sets the stage for preventive strategies of certain types of cancer. The idea is to either manage or eradicate the small cell populations that constitute incipient lesions. Ideally, the size of the population to be targeted would be a single transformed cell, but thousands or tens of thousands cells could still be acceptable, although the upper limit is not known for any cancer. Put simply, if incipient lesions have low degrees of heterogeneity, they are less likely than full-grown tumours to contain treatment-resistant cells. It follows that low therapeutic doses of appropriate drugs should not only eliminate early cancers but also would not select for cells resistant to high chemotherapeutic doses, as the (epi)genetic variants compatible with resistance are less likely to have already emerged. Thus, while the risk of cancer developing years or tens of years following the treatment is expected to be reduced, if low-dose preventative therapeutic intervention was to fail, then the unverified prediction is that those malignancies that do eventually emerge will be no more resistant to chemotherapies than would have been the case had the preventive treatment not been administered. This crucial hypothesis will need to be carefully tested in animal models before large-scale preventive intervention is envisaged. However, some supportive evidence already exists. For example, although the preventive efficacy of tamoxifen for women who are BRCA 1 or 2 carriers is controversial, to the best of our knowledge no increase in drug-resistant tumours has been reported for this chemopreventive strategy (Metcalfe and Narod 2007). Furthermore, a recent clinical trial of continuous versus intermittent androgen deprivation treatment for patients postradiotherapy for localized prostate cancer, while showing no benefit on overall survival for either group, indicated that the intermittent cohort had a statistically significant lower risk of developing an androgen-independent disease (Crook et al. 2012). This could be interpreted as suggesting that the periods without treatment gave a competitive advantage to drug-sensitive cells, which *in fine* decreased the emergence of fully resistant tumours.

We suggest that for individuals in specific high-risk groups, but with no apparent symptoms and thus clinically healthy, there is a high benefit/risk ratio for using low doses of therapies with minimal side effects aimed at a single (epi)genetically changed clone that has a high probability of leading its descendants down the path of tumourigenesis (see Wu and Lippman 2011; for focus on minimizing toxicity; Maley et al. 2011; for a related perspective on cancer prevention). An alternative preventive approach would be to focus on alterations of glucose or lipid metabolism, characteristic of all tumours (Porporato et al. 2011; Dang 2012). Yet another strategy is to target tissues in the tumour microenvironment and/or microinflammation (e.g. Gatenby and Gillies 2008; Gatenby 2011; Gillies et al. 2012; Rothwell et al. 2012). For example, the control of local acidosis in premalignant lesions was recently shown to significantly increase overall survival in mice treated early in tumour progression (Ibrahim-Hashim et al. 2012).

We call sets of measures rooted in evolutionary theory and designed to prevent disease status or to manage or eradicate incipient disease-causing organisms or cells *preventive evolutionary medicine*. Preventive evolutionary medicine can take the form of either changes in lifestyle so as to either maintain or bolster natural defences, or more specific, active treatments, designed to prevent the initiation, growth, spread and resistance status of an infectious or noninfectious disease-causing agent. Perhaps the best-known example of preventive evolutionary medicine is vaccination and there is a major effort to develop prophylactic and therapeutic vaccines aimed at cancer causing pathogens and/or cancerous cells (e.g. Finn 2003; Palucka et al. 2011). However, the impressive success of vaccination in the control of many infectious diseases notwithstanding, there is evidence that infectious agents can evolve in virulence and transmissibility when confronted with protected or partially protected hosts (Gandon and Day 2008). While we do not know to what extent the evolution of resistance to specific treatments may occur in cancer prevention, as mentioned above, there is a risk that aggressive metastatic mutants may be selected by low-dose preventive therapies, for example, due to alteration of the microenvironment (Albini and Sporn 2011).

## Examples of targeted therapies

For preventive approaches to be successful for cancer, therapies will need to have effects targeted either directly or via the microenvironment, to the self-renewing cell types that serve as progenitors to malignancy. All aggressive tumours have common characteristics, the so-called cancer hallmarks (Hanahan and Weinberg 2011). They include unlimited replicative potential, resistance to programmed cell death, a degree of independence from the environmental cues for growth and cell division, altered metabolism, stimulating angiogenesis, escape from immune responses and, for metastatic tumours, acquisition of cell motility and invasiveness. By definition, early (pre)cancerous lesions do not display all of the hallmarks. Unfortunately, despite some early hope of defining the precise sequence of events leading to tumour emergence (Vogelstein and Kinzler 1993) and theoretical studies suggesting pattern in the temporal sequence of hallmark acquisition (Sprouffske et al. 2011), it would seem that acquisition of almost any hallmark might be the first step in tumour initiation and a target for prevention. For instance, changes in cell morphology, generally considered to precede acquisition of cell motility (Wodarz and Nathke 2007; Akkari et al. 2012), give rise to dysplasia, characteristic, for example, of hepatocellular carcinoma, the most frequent primary liver cancer. Metaplasia, which shows independence from environmental control signals, is a characteristic of a precancerous condition of Barrett's oesophagus despite its possible physiological adaptive advantage (Reid et al. 2010; Wang et al. 2011), while hyperplastic lesions, due to excessive proliferation, are observed prior to colon carcinoma (Jones et al. 2008). Resistance to cell death through overexpression of Bcl2 antiapoptotic protein is probably an initiating event of a large majority of follicular lymphomas (Tsujimoto et al. 1987). Importantly, all the hallmarks are controlled by a large number of molecular pathways, each composed of many molecular actors. Some pathways are frequently deregulated in specific tumour types, such as EGFR/Ras/MAPK (Mitogen-activated protein kinase) signalling in lung or in colorectal carcinogenesis (Cejas et al. 2012). However, even in these apparently clear-cut cases, which are good candidates for targeted personalized treatment, the culprit signalling pathway can be altered at different levels, for example, through the expression of a mutated upstream growth factor receptor (EGFR) or its downstream effector (K-Ras). Unfortunately, a molecular diagnosis of newly formed sporadic precancerous lesions is likely to be very challenging if not impossible. However, whatever other alterations a precancerous lesion or a newly emerged neoplasm might have acquired, to constitute any threat, it must be characterized by a growth advantage. Thus a preventive strategy could be to use low levels of low-specificity, antiproliferative treatments to either target reductions in cancer cell proliferation or shift the competitive balance in favour of healthy cells, for example, through interference with oncogene-driven cell competition. The complex process of active cellular competition was originally described in Drosophila, where ‘winner’ cellular clones eliminate the ‘losers’ through active signalling, unrelated to their intrinsic proliferative capacity. This was recently confirmed in mammalian cells and is suspected, albeit not proven, to participate in tumour cell dynamics (Maley et al. 2004; Johnston 2009).

The picture is quite different for individuals with a strong genetic predisposition to cancer. For example, mutations in BRCA1 and 2 genes, which code for proteins involved in the process of DNA repair, have an overall prevalence in human populations of 1/400 and 1/800, respectively, and considerably higher in specific ethnic groups. The penetrance, that is the lifelong cumulative risk of breast or ovarian cancer for women with germline heterozygous mutations in either of these genes, is 40–60% (Petrucelli et al. 2010). A remarkable recent development in the treatment of cancers harbouring mutant BRCA is the use of poly-ADP ribose polymerase (PARP) inhibitors. PARP is an enzyme that acts in a parallel DNA damage repair pathway to that governed by BRCA (de Murcia and Menissier de Murcia 1994). Whereas inhibition of either of these DNA repair mechanisms is compatible with cell survival, eliminating both gives rise to so-called synthetic lethality (Dobzhansky 1946) by efficiently preventing the cell from counteracting accumulating DNA lesions. Several PARP inhibitors are currently under clinical trials for advanced breast and ovarian cancer, giving good objective response rates and limited adverse effects (Tutt et al. 2010). However, as is the case for all molecularly targeted therapies currently available for solid tumours, patients almost always relapse (see e.g. Le Cesne et al. 2010).

Given our current understanding of the underlying biology and the extremely high risk of cancer for individuals carrying a mutant BRCA allele, there is some urgency to undertake both preclinical and clinical tests for preventive therapy based on PARP inhibitors, most probably in association with additional drugs, such as cisplatin or topoisomerase I inhibitors, already used in combination therapies (Zander et al. 2010; Rottenberg et al. 2012).

## Potential for (and limitations of) prevention

Despite the widespread awareness of the importance of cancer prevention, the logic of active preventive evolutionary medicine has received less attention compared with curative strategies. In addition to alteration of the environment (to reduce involuntary exposures to carcinogens) and changes in human behaviours (to reduce voluntary or inadvertent exposures), cancer prevention could take two main routes: reinforcing existing disease check mechanisms through, for example, prophylactic vaccination (Finn 2003), and attempting to manage or eradicate neoplasms before resistant strains emerge. The former process, which is distinct from the widely investigated, although so far rarely successful, immunotherapy of existing disease, aims to augment anticancer defences, thus amplifying what natural selection has limited power in achieving (Dunn et al. 2004). The second process is an ecological reinforcement of what evolution has taken hundreds or possibly many thousands of generations for human populations to put into place through adaptation. This route exploits what we already know about population size and the likelihood of the existence or emergence of resistance mutations. Resistant phenotypes are either already present at low frequencies in detectable neoplasms or emerge during treatment (Frank and Rosner 2012; Greaves and Maley 2012). If a treatment-sensitive tumour is genotypically homogeneous (e.g. mutation of *c-kit* or *PDGFR* in a gastrointestinal tumour), then it is unlikely that resistant cells will be present. Certain cancers, however, have well-characterized defects in the DNA repair processes and are known for their genetic variability at relatively small cell population sizes, for example, *BRCA* breast and ovarian tumours, Fanconi anaemia-associated cancers or HNPCC-associated colorectal tumours (Lengauer et al. 1998; Deans and West 2011; Loeb 2011). Interestingly for the two former cancers, the current targeted therapies push them towards an ‘error catastrophe’, as treatments further inhibit their capacity to repair DNA lesions, thus increasing their mutation load to the extent of making them nonviable and reducing tumour size (Ashworth 2008). In evolutionary terms, we can consider that the cost to cancer cells of acquiring the trait of genetic instability (through defective DNA repair) is the risk of attaining too much instability, resulting in cell death. The previously mentioned PARP inhibitors play just this role. However, a thin line separates ‘too far’ (lethal) from ‘just far enough’ (even higher genetic instability, but still compatible with cellular survival). While very careful investigation is required to determine if preventive approaches might not accelerate malignancies or create new ones in the background of genetic instability (and animal models are available for at least initial highly controlled preventive trials; van Miltenburg and Jonkers 2012), these genetic predispositions remain particularly attractive for a preventive evolutionary medicine approach.

What kinds of conditions are particularly amenable for preventive evolutionary medicine? It is estimated that 5–10% of cancers arise in the context of genetic predisposition, with variation between different tumour types ranging from less than 0.5% for connective tissue or testicular tumours to about 15% for prostate carcinoma (Hemminki et al. 2004). Doubtless, a much clearer picture will emerge from analyses of the massive data that are accumulating from next-generation sequencing. This will help define specific groups likely to benefit not only from personalized treatment (Mendelsohn et al. 2012), but also from novel preventive strategies, such as the ones discussed here. Although research aimed at defining personalized treatments for cancer patients is developing at an impressive rate, the number of drugs, be it small molecules or antibodies, actually available in the clinic is currently limited, with, for example, only 16 approved in France (Table 1). Until new, reliable targeted therapies are discovered, existing classical and personalized treatments continue to be explored in search of synthetically lethal combinations for different types of cancer (Garnett et al. 2012; Hoelder et al. 2012). While not yet validated in the clinic, there are reasons for optimism that numerous novel treatments will become available in the near future that can be used both for advanced disease and for early preventive strategies.

**Table 1:** Targeted therapies for solid tumours currently in clinical practice in France

DCI	Targets	Indication
**Anti angiogenics**		
Bevacizumab	VEGF	Renal carcinoma, Breast carcinoma, Colorectal carcinoma, Chest carcinoma. Glioblastoma
Sunitinib	VEGF-R, PDGF-R, c-Kit	Renal carcinoma, Thyroïd, Pancreatic endocrine tumor, Gastrointestinal stromal tumor
Sorafenib	VEGF-R, PDGF-R, c-Kit Raf	Liver carcinoma, Renal carcinoma
Regorafenib	VEGF-R, PDGF-R, c-Kit, FGF-R Raf	Colorectal carcinoma
Pazopanib	VEGF-R, PDGF-R, c-Kit	Renal carcinoma
**Anti HER 1/HER 2**		
Lapatinib	EGF-R HER2	Breast carcinoma
Trastuzumab	HER 2	Breast carcinoma, Gastric carcinoma
Erlotinib	EGF-R	Chest carcinoma, Pancreatic carcinoma
Gefitinib	EGF-R	Chest carcinoma
Cetuximab	EGF-R	Colorectal carcinoma, Head and neck carcinoma
Panitunumab	EGF-R	Colorectal carcinoma
**mTOR Inhibitor**		
Temsirolimus	mTOR	Renal carcinoma
Everolimus	mTOR	Renal carcinoma, Endocrine tumor, Breast carcinoma
**Others**		
Imatinib	c-Kit	Gastrointestinal stromal tumor
Olaparib	PARP	Ovarian cancer
Vemurafenib	Raf	Melanoma

VEGF, vascular endothelial growth factor; PDGF, platelet-derived growth factor; EGF, epidermal growth factor; HER, human epidermal growth factor receptor; mTOR, mammalian target of rapamycin; PARP, poly(adenosine diphosphate [ADP]–ribose) polymerase.

As for all large-scale public health strategies, targeted chemoprevention of cancer would be costly. While detailed prediction of the monetary cost is beyond the scope of this work, considerations for such an assessment should include (i) the number of treatments, doses and products employed for preventative therapy versus analogous interventions once a cancer is detected (including costs associated with possible cancer relapse), (ii) over treatment, that is the percentage of people treated preventatively who would not have developed cancer at a later time and (iii) costs related to decreased capacity of work of cancer patients. These costs do not include other important considerations that are difficult to quantify monetarily, in particular (i) the psychological (collateral) effects of preventative treatments versus cancer diagnosis and associated therapy and (ii) lifestyle benefits of successful prevention.

Before such an in-depth economic analysis is performed, we note that about 10% of all cancers arise in genetically ‘high risk’ groups, which represent less than 1% of the total population. The current cost of treatment of these patients in the USA is a staggering $15 billion per year (Mariotto et al. 2011). While this is above the expected cost of low-dose preventive therapy for people at risk, whether large-scale chemoprevention becomes economically viable will depend on its success rate: that is the decrease, or at least significant delay, in the occurrence of cancers in the preventively treated group.

## A conceptual framework

**Figure 2:**
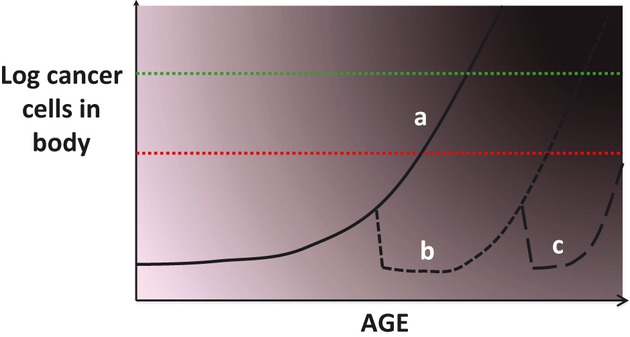
Framework for preventive cancer therapy. This hypothetical graph shows the probability of mortality (shading level) as a function of a person's age and number of cancer cells in the body. Lower horizontal line represents the tolerance threshold in the cancer cell population, beyond which there is an unacceptably high likelihood that chemoresistant cells will be present. This threshold will vary from person to person, and depend on cancer type, the tumour microenvironment and (epi)genetic instability. Determination of the tolerance threshold and an understanding of cancer cell demography will form the basis of a predictive framework for the timing of one or more therapeutic interventions, with the objective of providing lifelong chemosensitivity and the control of cancer cell number to nonharmful levels. Interventions not only reduce cancer cell populations, but also reduce (epi)genetic variation, which are the source of the evolutionary emergence of chemoresistant lines. The upper horizontal line is the cell density beyond which tumour detection is likely. Case a: no therapy. Case b: single therapy that alone cannot prevent eventual chemoresistance and mortality from the disease. Case c: a second therapeutic treatment, resulting in no resistance and in population control.

**Figure 3:**
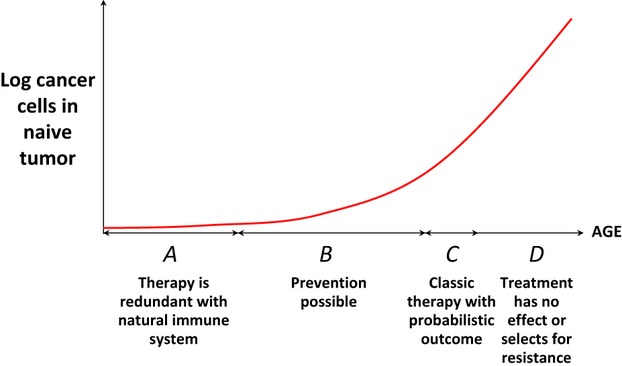
Windows of opportunity for different therapeutic approaches. (A) Therapy redundant with natural control; (B) Preventive therapy possible due to low malignant cell number and few or no chemoresistant clones; (C) Classic therapy following cancer detection gives a probabilistic outcome as chemoresistant clones in the malignant neoplasm are very likely; (D) Metastatic disease with low probability of curative success; low-dose palliative approaches may extend life.

Figure 2 presents a conceptual framework for evolutionary preventive medicine of cancers. For people at high risk of emergence of cancerous states due to genetic predisposition or exposure to an environmental hazard (e.g. certain chronic infectious diseases, high radiation levels), preventive treatments would need to be periodic (curves B and C in Fig. 2). However, cancers with long lag times (e.g. lung or throat cancers due to tobacco) present large windows of opportunity for killing incipient lesions, suggesting that preventive treatments might only need to be administered once or a small number of times during a patient's life. The width of such windows of opportunity (Fig. 3) will evidently be characteristic for each cancer type (and probably levels of carcinogenic exposure) and some cancers will have greater variability from person to person than others. Indeed, it is likely that cancers with excessively small or highly variable windows will not be conducive to preventive medicine. Theoretical study employing a recent mathematical model and associated parameter estimates based on Bozic et al. (2010) indicates that the window of opportunity before the first chemoresistant mutation emerges is typically 10 years or more, and the number of cells in the neoplasm at the time of emergence may be as little as 100 (Hochberg, unpublished numerical studies). Additional mathematical analyses combined with laboratory experiments will be necessary to produce a predictive, operational theory for cancer-specific and perhaps patient-specific preventive evolutionary medicine (see also Komarova and Wodarz 2005).

## Future directions and conclusion

Evidently, many people already do alter their lifestyles with the aim of preventing different types of disease, including a variety of modifications with more or less proven efficiencies to reduce the risks of contracting cancers (ceasing to smoke, reducing alcohol consumption, exercising, increasing the consumption of antioxidants, immune system reinforcement, etc.). We need to better understand how lifestyles and their changes influence evolutionary cell biology, and in so doing, change cancer dynamics. To the extent that actual medical intervention is possible for certain cancers, we need to evaluate how molecules, either with specific actions on cells (e.g. inducing selective apoptosis, dormancy) or presumably with a systemic effect on the microenvironment (e.g. aspirin) affect the ecology and evolutionary dynamics of the transition from benign to malignant neoplasms. However, it is necessary to keep in mind that there are many valid critiques for the development of specific molecules to prevent cancers. As we have argued above, these include health programme costs, side effects and whether such drugs risk inducing new cancers or other diseases. Similarly, because different anticancer strategies (tolerance/avoidance versus resistance/eradication) that our bodies naturally employ are ostensibly a product of our evolutionary history (Fig. 1; see e.g. Read et al. 2008), artificially targeting incipient cancers could create novel environments for healthy cells and possible unexpected treatment side effects, such as selection for chemoresistance. Given these important concerns, we stress that due caution needs to be exercised in the development and application of any preventative approach.

Moreover, potential side effects will mean that the notion of disease ‘risk’ must be integrated into decision making if the idea of preventive evolutionary medicine for cancer is to gain any traction. Such disease ‘risks’ include genealogical factors or genetic predispositions to the disease, elevated exposure to carcinogens including cancer-causing pathogens and previous bouts with cancer. Preventive therapies directed at cells (as opposed to tumour microenvironments) would need to be cancer cell specific, targeting either known genetic defects, such as PARP inhibitors for patients at high cancer risk due to mutations in BRCA1/2, ATM or PTEN genes (Chalmers et al. 2010), environmental exposures such as oestrogen for ER+ breast tumours (Althuis et al. 2004) or interference with cancer-specific metabolic requirements (e.g. Porporato et al. 2011).

We have a scientific understanding of why current approaches to cancer eradication generally fail: chemotherapies select resistant cells and these are likely to grow unfettered during and possibly post therapy. Although we obviously must continue to investigate declared cancers with new and innovative methods, we argue here that we also need to carefully consider active, interventionist strategies that prevent cancers in the first place or eradicate them prior to detection, especially given the likelihood that we are all continuously, naturally controlling precancerous lesions. For some types of cancer and for those individuals at heightened risk of contracting the disease, we propose that this natural control could be supplemented with preventive measures, based on insights from evolutionary biology.
